# Prevalence of Penicillin Resistance Among Streptococcus pneumoniae Isolates in a General Hospital in Southwest Saudi Arabia: A Five-Year Retrospective Study

**DOI:** 10.7759/cureus.55326

**Published:** 2024-03-01

**Authors:** Muhammad Halwani

**Affiliations:** 1 Department of Medical Microbiology, Faculty of Medicine, Al Baha University, Al Baha, SAU

**Keywords:** vaccination, saudi arabia, retrospective study, penicillin-resistant, streptococcus pneumoniae

## Abstract

Background

The rise in infections caused by penicillin-resistant strains of *Streptococcus pneumoniae* has become a global concern. However, the magnitude of this problem in Southwest Saudi Arabia has never been investigated. Therefore, this study aims to determine the prevalence of this bacteria in the region using in vitro data.

Materials and methods

This study retrospectively studied pneumococcal isolates collected by the Microbiology Laboratory of a general hospital in Al Baha, Saudi Arabia, from January 2013 to December 2017. A minimum inhibitory concentration (MIC) ≥ 8 mg/L was used as a cutoff concentration to detect the resistant isolates.

Results

A total of 201 S.* pneumoniae* isolates were identified using the VITEK® 2 system (bioMérieux SA, Marcy-l'Étoile, France). Most of these isolates (61%) were obtained from respiratory specimens, including sputum, tracheal aspirates, and bronchoalveolar lavage. Eye swabs accounted for 15% of the isolates, blood samples contributed 12%, ear swabs accounted for 7%, and cerebrospinal fluid (CSF) 3.4%. The resistance of *S. pneumoniae* during the five years varied from 61% to 76%, with an overall resistance of 70% (141/201). The resistance rate per year was 71% (43/60) in 2013, 76% (35/46) in 2014, 61% (22/36) in 2015, 68% (20/29) in 2016, and 66% (21/30) in 2017.

Conclusion

The data confirm the presence of penicillin-resistant *S. pneumoniae* in Southwest Saudi Arabia. Furthermore, the high resistance suggests a potential concern, emphasizing the need for penicillin control, surveillance, and vaccination to address this growing problem.

## Introduction

*Streptococcus pneumoniae* is a prevalent pathogen that poses a significant threat in terms of focal and systemic illnesses, particularly among children and older adults, leading to substantial morbidity and mortality [1,2]. This condition may worsen in countries where the pneumococcal vaccine is unavailable [3]. The World Health Organization (WHO), in 2014, considered pneumococcus as one of the nine bacteria of international concern [4]. The emergence of widespread resistance to penicillin by pneumococcus in various regions around the world has recently become a cause for concern [5-9]. This increasing prevalence of multi-drug resistant strains necessitates re-evaluating the empirical therapy for pneumococcal infections, highlighting the urgent need for better pneumococcal vaccines tailored to older adults and children.

The prevalence of antibiotic resistance in *S. pneumoniae* in Saudi Arabia has been studied in various regions [10-13]. However, it is essential to note that these studies primarily focused on the major cities of the country without adequately exploring the resistance patterns in the lesser populated regions like the southwest. Thus, this study aims to fill part of this gap by analyzing retrospective data from the microbiology laboratory in a general hospital in southwest Saudi Arabia to determine the prevalence of penicillin resistance among *S. pneumoniae* isolates in this part of the country.

## Materials and methods

This was a retrospective study conducted in the Microbiology Laboratory of King Fahad Hospital, Al Baha, Saudi Arabia. The study included microbiological data collected from the laboratory database for the period spanning January 2013 to December 2017.

Bacterial isolates data collection

Only the specimens that grew *S. pneumoniae* throughout the five years were collected and included in the study. Specimens were received in the hospital laboratory upon clinical requests. Routine identification of isolates was performed in the laboratory using standard laboratory identification methods. Bacteria suspected to be gram positive and exhibiting α-hemolytic reaction on Mueller-Hinton agar supplemented with 5% sheep blood were identified after the plates were incubated in 5% CO2 at 35⁰C for 20-24 hours.

Confirmation of the isolates

To confirm the identification of the grown bacteria, a pneumococci latex agglutination kit (Pneumotest-Latex) was employed, as stated by Slotved et al. [14]. Strains that showed a positive reaction, indicated by clumping of the latex particles within 10 seconds of the test performance, were considered *S. pneumoniae*.

Full identification and sensitivity testing

Identification up to the species level and simultaneous antibiotic susceptibility testing based on the minimum inhibitory concentration (MIC) of the identified strains were determined using VITEK® 2 system (bioMérieux SA, Marcy-l'Étoile, France) [15]. Resistance to penicillin was identified by the machine using the following criteria: susceptible < 2 μg/ml, intermediate < 4 μg/ml, and resistant ≥ 8 μg/ml, as described by Cilloniz et al. [16].

Data analysis

Numeric data was represented using simple tabulation, with a tabular format utilized to present numerical values and proportions across rows and columns. The initial data set encompassed details on specimen types, corresponding numbers, and their respective proportions. Subsequently, a second data set was compiled, comprising the total count of *S. pneumoniae* isolates identified, the proportion of isolates demonstrating resistance to penicillin, and finally breakdown of the number of isolates alongside the proportion exhibiting susceptibility to various antibiotics tested was presented in this data set. The rate of resistance was compared between the study years using a paired samples t-test. No attempt was made to investigate any clinical data (apart from the specimen types) or treatment response on the relevant patients and only in vitro information were gathered in this study.

## Results

Over the course of five years, a total of 201 *S. pneumoniae* isolates were identified, with 61% (n=124) obtained from respiratory specimens, including sputum, tracheal aspirates, and bronchial lavage fluid. Additionally, 15% (n=30) of isolates were obtained from eye swabs, 12% (n=25) from blood, 7% (n=15) from ear swabs, and 3.4% (n=7) from cerebrospinal fluids (CSF) (Table 1).

**Table 1:** Specimen type and total number of Streptococcus pneumoniae isolates identified.

Specimens	Number	Rates (%)
Respiratory (sputum, tracheal aspirates, bronchoalveolar levage *)*	124	61
Eye swabs	30	15
Blood	25	12
Ear swabs	15	7
CSF	7	3.4
Total	201	100

**Figure 1:**
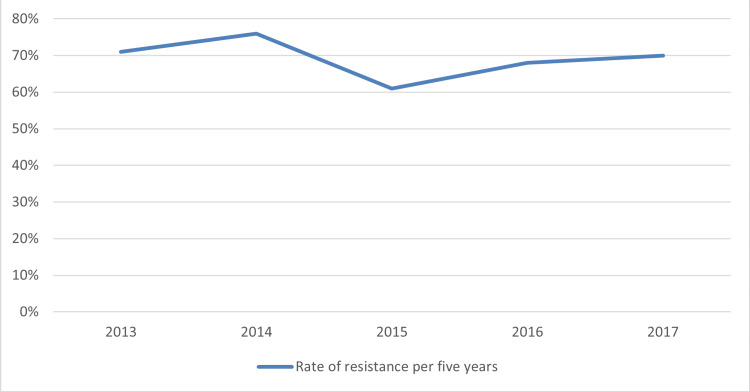
Rate of resistance (%) of Streptococcus pneumoniae isolates to penicillin 10µ during the five years. A minimum inhibitory concentration (MIC) ≥ 8 mg/L was used as a cutoff concentration to detect the resistant isolates

The resistance rate to penicillin for the entire five-year period was 70% (n=141) and ranged from 71% in the year 2013 to 66% in the year 2017, as shown in Figure 1, but no significant differences in resistance rates were observed between the first year and the final year of the study after using paired samples t-test (P = 0.94). 

In the context of antibiotic susceptibility testing, vancomycin emerged as the most effective antibiotic against the strains under test, with a 100% rate of susceptibility. This finding was supported by clindamycin, which demonstrated a lower, yet significant, susceptibility rate of 78%. Erythromycin and cotrimoxazole exhibited moderate susceptibility rates of 54% and 40%, respectively, while cephalothin displayed the lowest susceptibility rate among the tested antibiotics at 33%. These findings are presented in Table 2.

**Table 2:** The most effective antibiotics against Streptococcus pneumoniae isolates (N=201)

Antibiotic	Number of sensitive isolates	Rate (%)
Vancomycin	201	100
Clindamycin	158	78
Erythromycin	112	54
Cotrimoxazole	81	40
Cephalothin	67	33
Penicillin	60	30

## Discussion

This study supports evidence from previous research by Bogaert et al. [17] and Deng et al. [18], who postulated that *S. pneumoniae* is more commonly involved in respiratory tract infections than other types of infections. In the present study, about 61% of collected specimens were from lung and airway secretions, indicating this organism’s high involvement in respiratory tract infections. This may suggest a probable correlation between the nasopharyngeal carriage of *S. pneumoniae* and the occurrence of respiratory tract infections, as reported elsewhere [19]. However, due to the focus of the current study as an in vitro study, it was not possible to definitively confirm these findings and investigate the clinical complications.

One unanticipated discovery in this study was the high prevalence of *S. pneumoniae* in eye swabs, accounting for 15% of the specimens collected. This is noteworthy since eye swabs are not typically considered a primary source for isolating this organism. Previous research by Mohamed et al. reported the increased involvement of *S. pneumoniae* in children younger than 24 months [20]. The present study had isolates from both adults and children; therefore, it is difficult to determine the group from which these positive isolates originated. Additionally, it is common practice for treating clinicians to delay prescribing antibiotics for eye infections to minimize their unnecessary use and rule out viral infections. Therefore, the unexpectedly high rate of positive eye swab specimens for culture and sensitivity in this study was not anticipated, as highlighted by Everitt et al. [21].

Bloodstream infection (BSI) caused by *S. pneumoniae* accounted for only 12% of the total isolates identified in this study, which is relatively low compared to other studies on the same organism [22,23]. However, this finding can be contradicted. Firstly, the isolates identified in this study were collected from different patients across various departments, including intensive care units, and different age groups. Additionally, this study did not investigate any specific patient risk factors. Therefore, it is currently unclear whether the low number of *S. pneumoniae* BSI cases identified in this study is due to a low overall prevalence of the organism in the hospital or if other factors contributed to *S. pneumoniae* being less predominant in the collected blood samples.

Ear infection with *S. pneumoniae* accounted for 7% of the total isolates collected. The current study's results have a much lower rate of isolation compared to that of Horhat et al. [24], who found that *S. pneumoniae* was identified in 35.7% of the patients studied, which led to certain severe complications like mastoiditis. Similarly, Zielnik-Jurkiewicz and Bielicka [25] reckoned that *S. pneumoniae* reported for 65.4% of the strains identified in their study. Both studies clearly showed that *S. pneumoniae*, particularly the penicillin-resistant ones, can lead to severe ear infections and treatment failure. Although, in this study, *S. pneumoniae* accounted only for 7% of the total isolates identified from ears, the severity of the infection and its clinically associated complications may not be far from those reported in previous studies.

Lastly, only 3.4% (7/201) of *S. pneumoniae* isolates were identified from the CSF. Although the frequency of identification in the CSF was low compared to other specimen types, the presence of *S. pneumoniae* in CSF can still pose severe complications, specifically meningitis [26]. Furthermore, even with treatment, complete recovery may not be achievable [27]. These findings underscore the potential seriousness of *S. pneumoniae* infection when it invades the meninges, particularly in cases where there is a delay in initiating appropriate antibiotic therapy or selecting the right antibiotic empirically [28]. However, as stated earlier, this study was conducted in vitro and did not directly observe associated complications or complete recovery.

The resistance of *S. pneumoniae* to penicillin, which used to be the drug of choice for a long time [29], is considered a global problem that is dramatically increasing [30,31]. In Saudi Arabia, the problem is much the same as penicillin- resistance *S. pneumoniae* has been reported in different regions including Riyadh by Shibl et al. [32], Al-Aqeeli et al. [10], and Twum-Danso et al. [33], Dharan by Al-Tawfiq [34], and Jeddah by Eltahawy [35]. Nevertheless, there has been a lack of extensive research focusing on the Al Baha region of Saudi Arabia. Hence, this study aims to provide the first investigation of penicillin resistance in this specific area of the country using in vitro data. The findings of this study are consistent with previous research in other regions of Saudi Arabia in detecting penicillin resisyance in *S. pneumoniae*. However, the overall rate of penicillin resistance observed in this study, reaching 70%, is notably higher than in previous studies conducted in the country.

The survey conducted in Jeddah, West Saudi Arabia, which is not very far from the Al Baha area, by Eltahawy [35] clearly stated that 53% of the pneumococcal strains identified in a two-year period were penicillin resistant. The researcher also predicted the potential spread of resistance to other regions of the country. Al Baha, situated in southwest Saudi Arabia, is known for its favorable weather conditions, particularly during the summer, attracting visitors from various parts of the country. This influx of people from different regions may contribute to the transmission of penicillin resistance in the area, as previous studies have confirmed person-to-person transmission of this organism during close contact [36]. Unfortunately, it was not possible to determine whether the identified isolates in this study were from residents or visitors. Therefore, caution must be taken when interpreting these results. Although this study focuses only on in vitro data, many risk factors related to penicillin resistance in *S. pneumoniae *were identified in the literature that could explain such a finding. The use of a β-lactam antibiotic in the previous three to six months has been confirmed as a risk factor [37,38]. A strong association has been identified between heavy penicillin use and the increase in resistance [39], which may also be the case in this study. Additionally, it has been found that around 20-50% of the healthy community population, either under five years or over 65 years of age, is colonized with resistant strains of *S. pneumoniae* [40]. All that factors listed above could have contributed either individually or combined to the findings of the current study.

In this study, in 2013, 71% (43 out of 60) of cases exhibited resistance to penicillin; however, in 2017, the resistance rate decreased to 70% (21 out of 30). The numerators showed differences between the two years, the first and the last year of the study. However, no statistically significant differences were observed when comparing the resistance rates between these periods (P = 0.94). This indicates no change in resistance rates from 2013 to 2017, although the nominators varied. In fact, the resistance rate was constant throughout the five years. These high resistance results corroborate the findings of a recently published work where the majority of the isolates, 98.9%, were completely resistant to penicillin [41].

It is noteworthy that until 2018, antibiotics could be obtained without prescriptions in Saudi Arabia, leading to widespread misuse. An investigation conducted in Saudi Arabia in 2017 found that 48% of the study population (473 persons with a mean age of 27 years old) obtained antibiotics without prescriptions through various means, such as purchasing them directly from a pharmacist based on his advice, self-prescribing based on previous expertise, or sharing with family members or friends [42]. This issue likely contributed significantly to the study's outcome. Fortunately, the Ministry of Health in Saudi Arabia implemented a new policy in April 2018, prohibiting the sale of antibiotics without prescriptions in pharmacies. As a result, over-the-counter antibiotic sales decreased significantly by 77%, as reported by Al-Tannir et al. [43]. This positive decision could contribute significantly to reducing antibiotic misuse in Saudi Arabia and most probably the emergence of resistance.

The second antibiotic with a high susceptibility rate is clindamycin, with around 78% of the isolates tested being susceptible. Although the resistance to clindamycin is not high in this study, it has been previously reported, and some isolates of *S. pneumonia* showed different grades of resistance [44]. A comprehensive review study found that in the United States, 65% of *S. pneumonia* isolates tested were resistant to clindamycin; however, the rate reached 90% in Europe [45]. This finding might also indicate that the use of clindamycin in this region of Saudi Arabia is less compared to other countries. It can be assumed that the global prevalence of macrolide resistance in *S. pneumoniae* has witnessed a recent surge, primarily linked to the widespread utilization of macrolides for treating respiratory tract infections acquired within different communities. Erythromycin, cotrimoxazole, and cephalothin were the other three antibiotics tested, and all had low susceptibility rates, which were 54%, 40%, and 33%, respectively. Different reasons can be responsible for this low susceptibility. However, a detailed discussion of these reasons is beyond the aim of the study.

As with all studies, this study has some limitations. Firstly, the study was conducted in a single hospital, which may limit the generalizability of the findings to other healthcare facilities. However, it should be noted that this hospital serves patients from various cities in Al Baha. It can be seen from the data in Table 2 that the panel of antibiotics tested in this study were penicillin, vancomycin, clindamycin, erythromycin, cotrimoxazole, and cephalothin. However, it must be stated that through the five years, some antibiotics were added, and some were missed or replaced. Only the antibiotics present throughout the five years were presented in this study. Vancomycin could be preferred with all tested isolates showing full sensitivity, aligning with previous studies [35,46]. Fortunately, vancomycin remains effective and can be used in case of invasive infections with resistant *S. pneumoniae* [41]. However, tolerance to vancomycin in *S. pneumoniae* has been previously reported in another study [47]. A possible explanation for this might be the overuse of the drug in that country compared to Saudi Arabia since these findings were not reported in any of the studies done in Saudi.

Moreover, the sample size was relatively small, particularly given the low prevalence of *S. pneumoniae*. While the study utilized a five-year data set, further research with larger sample sizes may be necessary to provide more conclusive results. Thirdly, the study did not distinguish between infection and colonization and did not categorize the study population based on age. Both factors could impact the interpretation of the results, as there may be differences in antibiotic resistance patterns between adults and children.

Also, the study did not explore the clinical outcomes associated with *S. pneumoniae* infections, making it unclear whether patients responded to antibiotic treatment. Furthermore, this study was limited to molecular investigations, which were not conducted on the identified isolates. Consequently, it remains unknown whether the isolated strains belong to the same genotype or are different in origin, as some studies on the same organism have reported distinctive serotypes [48]. Finally, since the vaccination against *S. pneumoniae* has begun in Saudi Arabia, the resistance rate might be decreasing. This study was conducted before the initiation of vaccination, and vaccination might have helped decrease the spread of resistant strains of *S. pneumoniae*.

Further research is needed to address these limitations and better understand the clinical implications of antibiotic resistance in this region. Future studies could focus on expanding the sample size, distinguishing between infection and colonization, categorizing patients based on age, and investigating clinical outcomes associated with *S. pneumoniae* infections. Additionally, molecular analysis could be conducted to better understand the spreading genotypes of *S. pneumoniae* in the area and compare these genotypes with those identified across the country.

## Conclusions

This study provides valuable insights into the prevalence and antibiotic resistance patterns of *S. pneumoniae* in the Al Baha region of Saudi Arabia. The findings support the global concern regarding the increasing resistance of *S. pneumoniae* to penicillin, with a notably high resistance rate of 70% observed in this study. The emergence of resistance may be attributed to factors such as overuse of antibiotics, lack of prescription regulation, and person-to-person transmission. Additionally, the study highlights the importance of vancomycin as an effective treatment option for invasive infections with resistant *S. pneumoniae*. Despite the limitations of the study, including small sample size and lack of clinical outcomes data, further research is warranted to address these shortcomings and explore the impact of vaccination on reducing the spread of resistant strains. Overall, these findings emphasize the need for continued surveillance and proactive measures to combat antibiotic resistance in the region.
